# HORMESIS RESULTS IN TRADE-OFFS WITH IMMUNITY

**DOI:** 10.1111/evo.12453

**Published:** 2014-06-20

**Authors:** Colin D McClure, Weihao Zhong, Vicky L Hunt, Fiona M Chapman, Fiona V Hill, Nicholas K Priest

**Affiliations:** 1 Department of Biology and Biochemistry, University of BathBath, BA2 7AY, United Kingdom

**Keywords:** *Drosophila melanogaster*, ecological immunity, fitness, hormesis, life-history evolution, trade-offs

## Abstract

Many have argued that we may be able to extend life and improve human health through hormesis, the beneficial effects of low-level toxins and other stressors. But, studies of hormesis in model systems have not yet established whether stress-induced benefits are cost free, artifacts of inbreeding, or come with deleterious side effects. Here, we provide evidence that hormesis results in trade-offs with immunity. We find that a single topical dose of dead spores of the entomopathogenic fungus, *Metarhizium robertsii*, increases the longevity of the fruit fly, *Drosophila melanogaster*, without significant decreases in fecundity. We find that hormetic benefits of pathogen challenge are greater in lines that lack key components of antifungal immunity (*Dif* and *Turandot M*). And, in outbred fly lines, we find that topical pathogen challenge enhances both survival and fecundity, but reduces ability to fight off live infections. The results provide evidence that hormesis is manifested by stress-induced trade-offs with immunity, not cost-free benefits or artifacts of inbreeding. Our findings illuminate mechanisms underlying pathogen-induced life-history trade-offs, and indicate that reduced immune function may be an ironic side effect of the “elixirs of life.”

Can organisms have it all? One of the central principles of life-history theory is that, because they are constrained by resource limitations, organisms cannot simultaneously optimize all aspects of fitness (Kirkwood 1977; Stearns 1992; Zera and Harshman 2001; Roff 2002; Zuk and Stoehr 2002). This premise has been challenged by studies reporting positive genetic correlations between fitness traits (Spitze 1991; Reznick et al. 2000; Hutchings 2006; Brzek and Konarzewski 2007; Koenig et al. 2009; Schroderus et al. 2012), studies revealing that longevity and fecundity can be decoupled with molecular genetics (Flatt 2011; Kenyon 2011), and studies documenting hormesis, which occurs when low doses of stress-inducing physiological treatments, such as heat shock, diet composition, and toxic chemicals, enhance traits associated with fitness (Minois 2000; Merker et al. 2001; Cypser and Johnson 2002; Hercus et al. 2003; Calabrese 2005; Gems and Partridge 2008; Hunt et al. 2011).

Research on hormesis demands attention from an evolutionary perspective (Forbes 2000; Costantini et al. 2010). Although it is not yet clear whether hormesis acts on Darwinian fitness, thousands of studies have documented the beneficial influence of stressors on important fitness traits, including longevity and fecundity (Calabrese 2005; Gems and Partridge 2008). Studies identifying beneficial influences of stress on fitness would challenge our understanding of evolution because it would imply that life histories are generally suboptimal (Forbes 2000). Still, with a handful of exceptions (Maynard Smith 1958; Krebs and Loeschcke 1994; Lane et al. 1996; Markowska 1999; Le Bourg et al. 2000; Sørensen et al. 2007), few studies have tested whether physiological treatments that extend life come at a cost to other aspects of life history, particularly in the ability to fight off live infections.

There are two lines of evidence implicating a link between hormesis and immunity. First, although parasites usually reduce the reproductive output and survival of their hosts (Lehmann 1993), a growing body of work shows that animals challenged with dead or even live pathogens exhibit improvements in specific aspects of their life history (Polak and Starmer 1998; Chadwick and Little 2005; Ikeda et al. 2007). In some animals, pathogen challenge increases resistance to subsequent infections (a pattern referred to as immune priming, see Pham et al. 2007; Lawniczak et al. 2007; Roth et al. 2009); but, in other animals, it enhances aspects of physiology, often to the detriment of their ability to fight off subsequent live infections (Leroy et al. 2012; Papp et al. 2012; Ermolaeva et al. 2013). Although it is unknown whether the physiological benefits of pathogen challenges fulfill the characteristic pattern of hormesis (an inverted “U” dose–response relationship with beneficial effects at low doses and toxic effects at high doses), the finding that life-history traits can be improved by a single dose of pathogen challenge suggests that hormesis can be induced by host responses to pathogen challenge (Leroy et al. 2012; Papp et al. 2012; Ermolaeva et al. 2013).

Second, even when the source of stress response is not a pathogen, hormesis appears to be driven by the expression of genes associated with immunity (Calabrese et al. 2012). Heat shock proteins not only contribute to heat shock induced increases in life span (Tatar et al. 1997; Kristensen et al. 2003), but also interact with components of the innate immune system (Chen and Cao 2010). Additionally, the NF-kB innate immune gene *Dif* has been shown to influence the hormetic benefits of cold shock (Le Bourg et al. 2012) and its expression is known to have a regulatory role in life-history trade-offs between longevity and immunity (Gosselin and Abbadie 2003; Rea et al. 2005; Mattson and Meffert 2006; Lemaitre and Hoffman 2007; Salminen et al. 2008; Pursall and Rolff 2011; Chirumbolo 2012; Gartner and Akay 2013). Other work indicates that the activation of innate immunity in response to pathogen challenge is linked both to enhanced physiology and reduced ability to fight off subsequent infections (Papp et al. 2012; Ermolaeva, et al. 2013).

Still, the evolutionary implications of hormesis are unresolved (Forbes 2000; Costantini et al. 2010). Does hormesis occur in outbred lines or is it only an artifact seen in nearly isogenic lines, as appears to be evident in diet-restricted, inbred mice (Liao et al. 2010)? Does hormesis represent a switch of life history, promoting survival in the detriment to other traits as suggested for calorie restriction (Tatar et al. 2003)? Does the expression of immune and stress genes generally facilitate or suppress enhancements in longevity? Does it make sense to recommend low-level stress as a therapy for human health, as some have done (Gems and Partridge 2008; Rattan and Demirovic 2009; Vaiserman 2010; Calabrese et al. 2012)? Or, does hormesis inevitably lead to trade-offs with immunity? These are important questions to resolve not only because they relate to how animals fight off infections and whether we can use stress treatments to improve health, but also because they provide a crucial test of the evolutionary principle that life-history optimization is constrained.

Here, we address these questions using the fruit fly, *Drosophila melanogaster* as host for the insect-generalist entomopathogic fungus, *Metarhizium robertsii* (for further details, see Gao et al. 2011; Zhong et al. 2013). This system has several features that make it suitable for studies of pathogen-induced fitness trade-offs and the age-specific genetic effects, which underlie hormesis: topical application of dead *Metarhizium* spores is known to stimulate immune responses in insects (Xia et al. 2001). Flies can be challenged by the fungal pathogen en masse by briefly agitating them in flasks with live or heat-killed fungal spores (Zhong et al. 2013). The fruit fly is well suited for large-scale experimental demography (Kohler 1994; Priest et al. 2002). By employing the *Drosophila* RNAi knockdown and mutant knockout lines in conjunction with appropriate control lines, we can assess the consequence of immune- and stress-response gene expression on longevity. Furthermore, we can also investigate the effects of pathogen treatment on life-history patterns in outbred laboratory lines to eliminate the possibility that hormesis is a side effect of inbreeding.

Our central hypothesis was that hormesis trades-off with immunity. This leads to the predictions that hormetic responses to stress should be greater in animals lacking functional immune responses and that hormesis should increase susceptibility to infection. To test these predictions, we used the following methodology: (1) we used an isogenic mutant stock of flies, *w1118*, to investigate the dose–response relationship between topical exposure to heat-killed fungus and resistance to heat stress. This established that a single dose of topical pathogen challenge was sufficient to induce hormetic benefits. (2) We studied a knockout mutant of *Hsp83* and used RNAi to down regulate three genes, *Dif*, *Turandot M*, and *Turandot C* (all derived from the *w1118* background), to test how the expression of immune and stress genes contributes to the fitness benefits of pathogen challenge. (3) Employing two outbred laboratory lines, we tested for pathogen-induced trade-offs between survival, fecundity, and susceptibility to subsequent live infections.

We chose the mutant and outbred laboratory lines for specific reasons. We investigated *Dif* because it is a key component of the Toll pathway, which confers antifungal immunity and is a putative regulator of hormesis (Le Bourg et al. 2012); *Turandot M* because it is upregulated in response to infection and provides protection against sexually fungal transmitted infections in flies (Ekengren and Hultmark 2001; Brun et al. 2006; Zhong et al. 2013); *Hsp83* as a positive control because previous work has established that stress-associated molecular chaperones are essential for hormesis (Tatar et al. 1997; Qin et al. 2005); and *Turandot C* because it is upregulated in response to many different types of stress and we had previously established that there was no evidence that it confers immunity to topical fungal infection (Zhong et al. 2013). But, findings in the aforementioned lines could be biased because they are mutant and are derived from an isogenic background. We therefore tested two wild-type lines, *Oregon-R*, a standard outbred laboratory-adapted line maintained in two-week culture (Milkman 1966), and *Dahomey*, another standard laboratory-adapted line maintained in large populations under age-independent culture (Chapman and Partridge 1996).

## Methods

### FLY AND FUNGUS STOCKS

All experimental animals were maintained at 25°C with 12:12 light:dark cycle in standard *Drosophila* shell vials at low densities (approximately 50 flies/vial) for three generations prior to the start of the experiments. We used an oatmeal-molasses-agar media with added live baker's yeast and an antifungal agent (Nipagin, Sigma-Aldrich, St. Louis, MO), which inhibits the growth of naturally occurring saprophytic fungi.

The *Dahomey* strain of *D. melanogaster* (obtained from Stuart Wigby, University of Oxford) was kept in large population cages (1 m^3^) with overlapping generations for two years before they were expanded over three generations in low-density culture (approximately 50 larvae/vial). The *Oregon-R* strain (obtained from Tim Karr, Arizona State University) was simultaneously expanded under low-density culture. We acquired the *Hsp83* knockout mutant, Act5C-Gal4 constitutive promoter, and *w1118* background strain on which the knockout and RNAi lines were based from the Bloomington Stock Center. The UAS-*TotM*, UAS-*TotC*, and UAS-*Dif* strains used were originally obtained from the Vienna *Drosophila* RNAi Center, which contained the RNAi constructs for the *Turandot M* and *C*, and *Dif* genes, respectively (for further information, see Dietzl et al. 2007; Zhong et al. 2013). The Gal4/UAS system operates by expressing the RNAi transelement for the target gene through the UAS promoter in all tissues of the fly, driven by the ubiquitous Act5C-Gal4 transcription factor providing universal knockdown of the gene.

We simultaneously generated nine distinct genotypes. We crossed Act5C-Gal4/CyO females with males carrying one of the UAS constructs to generate the genotypes with targeted gene knockdowns and a knockout: Act5C-Gal4/UAS-*TotM*-IR; Act5C-Gal4/UAS-*TotC*-IR; Act5C-Gal4/UAS-*Dif*-IR; and *Hsp83*^−^/*Hsp83*^−^. We crossed Act5C-Gal4/CyO females, UAS construct females, and *w1118* wild-type females to *w1118* wild-type males to generate control genotypes: +/+, Act5C-Gal4/+, UAS-*TotM*-IR/+, UAS-*TotC*-IR/+, UAS-*Dif*-IR/+. Thus, for each gene knockdown, there were three control genotypes (+/+, Act5C-Gal4/+, and UAS-*gene of interest*-IR/+), which permitted being able to account for independent effects of the Act5C-Gal4 promoter and UAS transgenes. The effectiveness of the knockdowns was confirmed by semiquantitative PCR (E. Immonen and M. G. Ritchie, unpubl. ms.). In total, we cultured 2592 vials of flies at 50 ± 10 larvae/vial before the start of the experiment (288 vials/genotype).

*Metarhizium robertsii* (isolate 2575) was obtained from the Agricultural Research Service Collection of Entomopathogenic Fungal Cultures (ARSEF, U.S. Department of Agriculture). This fungus is a common soil-associated, insect-generalist pathogen commonly used in pest control of large insects (Gao et al. 2011). We inoculated quarter-strength sabouraud dextrose agar with *M. robertsii* conidia (asexual fungal spores) and incubated the plates at 28°C for four weeks before storing at 4°C for up to three months. Conidia were collected by scraping the surface of the sporulating culture with an inoculating loop. Conidia were autoclaved by placing a large amount of live spores into a glass universal that was taped inside an autoclave bag. This ensured no moisture came into contact with the spores.

### METHOD OF PATHOGEN CHALLENGE

Each pathogen challenge treatment involved placing approximately 300 mixed-sex flies of each genotype without CO_2_ anesthesia into a 250-ml conical flask with 20 mg of autoclaved (heat-killed) conidia and agitating the flask for 10 sec. Exposed flies were held in temporary holding vials before being transferred to new food vials and into 10 × 15 cm demography cages (see Priest et al. 2002). This treatment method topically inoculates flies at fairly consistent doses of fungus, even after accounting for the effect of grooming on topical dose (Zhong et al. 2013). Sham-treated control flies were manipulated identically by agitating them in an empty flask. The procedure for testing susceptibility to live infection was identical, except that the conidia were not autoclaved before the treatment.

### INDUCING HORMESIS THROUGH PATHOGEN CHALLENGE

We first examined how the dose of pathogen challenge influenced resistance to heat stress, to determine whether pathogen challenge fits the inverted “U” (low-level, beneficial) pattern characteristic to hormesis. Flies of the *w1118* (+/+) genotype were collected from eclosion in a 24-h cohort and were left to mature in mixed-sex vials in densities of 50 flies/vial for two days. Following maturation, flies were placed on a 10-day regime where they were exposed to varying frequencies of pathogen challenge using the aforementioned method. The regime consisted of five treatments: 0—no pathogen challenge; 1—a single pathogen challenge on day 10; 1 *early*—a single pathogen challenge on day 2; 3—pathogen challenges on days 2, 6, and 10; and 5—pathogen challenges on days 2, 4, 6, 8, and 10. On days when flies on particular regimes were not exposed to a pathogen challenge, they were conditioned to sham treatment. This ensured that any responses observed were the result of the pathogen treatment. At day 12, all flies were conditioned to heat stress, 38.5°C for 45 min in a water bath, and survival was assayed 20 h posttreatment.

### EFFECTS OF PATHOGEN CHALLENGE ON LONGEVITY AND FECUNDITY IN THE W1118 BACKGROUND LINES

We used RNAi and mutant fly lines to assess the influence of stress and immune genes on the pathogen-induced responses on longevity. For each of the nine genotypes, flies were collected over 24-h cohorts, and held in mixed-sex cages. At day 7, following a randomized experimental design, the flies were either given a sham treatment or single dose of a pathogen challenge. Survival was assessed by recording and removing dead flies every two days post-treatment until all flies perished.

The fecundity of female flies was also assessed in each line to determine whether hormetic effects on longevity led to trade-offs with reproduction. Females were collected as virgins over a 4-h window (immediately subsequent to the original 24-h cohort) and placed in media vials at 20 flies/vial. After two days, 20 *w1118* +/+ males were added to each media vial to allow them to mate over a 24-h period. Twenty-four hours after the males had been discarded, females were given a pathogen challenge using the aforementioned method, except females were treated in groups of 20 flies with 6 mg of heat-killed spores. The females were then transferred to single-female food vials and subsequently transferred to fresh food vials every two days for a total of 10 days. Used food vials were held for 18 days after collection at 25°C and then frozen after which the number of hatched pupal cases was counted to estimate fecundity.

### TRADE-OFFS BETWEEN HORMESIS AND IMMUNITY IN WILD-TYPE LINES

We used two wild-type outbred fly lines, *Oregon-R* and *Dahomey*, to assess the influence of a single dose of pathogen challenge on survival, fecundity, and susceptibility to live infection. To assess survival, flies were collected over a 24-h period and matured for 48 h in mixed-sex cages. Female flies were given a pathogen challenge on day 4 and dead flies were removed and recorded every two days post-treatment when fresh media vials were supplied. Fecundity was assessed as above, except that the lines were provided males of their own strain and fecundity measures were taken over a total of four days.

To assess the influence of pathogen challenge on immunity, flies were collected over a 24-h period and held for two days in mixed-sex cages to ensure that they were mature prior to the challenge. Following maturation, flies were either given a pathogen challenge or a sham treatment. Two days post-treatment, all groups were treated with live fungal spores. Dead flies were recorded and removed daily following infection until all individuals in the vials were dead. Food was replaced daily.

### STATISTICAL ANALYSIS

We used chi-squared contingency tests to investigate the influence of the number of doses of topical treatment with heat-killed fungus on resistance to heat stress. Initially, the proportion alive following heat-stress was assessed across all treatment regimes. Following this, further analyses were completed to compare between individual treatments.

Cox proportional hazard regressions were used to analyze the influence of pathogen treatment on survival and to assess how the treatment responses differed between the genotypes. The full model included genotype, pathogen treatment, and pathogen treatment × genotype interaction as predictor variables, with age at death as the response variable considered with information on censoring (to account for the small number of flies that escaped during the study). ANOVAs were used to test the significance of interactions between predictor variables by comparing Cox regression models incorporating the interactions with models where they were removed. Separate Cox regressions were performed for each genotype and additionally for each gene of interest that only included the relevant knockdown and control genotypes (e.g., the analysis of *TotM* included the knockdown, Act5C-Gal4/UAS-*TotM*-IR, and the three control lines, Act5C-Gal4/+, +/UAS-*TotM*-IR, and +/+). For each gene of interest, we first estimated the hazard ratios (the change in the probability of death by the next event in pathogen-challenged animals relative to uninfected animals) for the knockdown genotype and also for its combined control genotypes (by pooling raw survival data of the relevant control genotypes) from the Cox models. Sequential Bonferroni corrections were completed on the significance values across the four comparisons. Percentage changes in survival were calculated by inverting the hazard ratio of the genotype to obtain the proportional difference in relation to controls. For heuristic purposes, mean longevity was also estimated for each genotype. A Pearson's correlation was used to assess concomitant changes in fecundity for all genotypes. Additionally, linear models were undertaken to identify the relationship between the effects on survival and fecundity for each knockdown/knockout genotype and their associated controls. The full model included the number of hatched pupae produced as the response variable and genotype, pathogen treatment, and pathogen treatment × genotype interaction as predictor variables. Sequential Bonferroni corrections were again completed across the four comparisons.

For the outbred wild-type lines, Cox regressions were completed for pathogen challenged relative to sham-treated animals when both infected and uninfected. Linear models were used to assess the fecundity of these populations with total pupae production as the response variable and treatment (pathogen challenged vs. sham treated) as a fixed effect. All analyses conformed to model assumptions and were performed with *R* version 2.15 (R Core Team 2012).

## Results

### A SINGLE DOSE OF TOPICAL PATHOGEN CHALLENGE ENHANCES RESISTANCE TO HEAT STRESS

**Figure 1:**
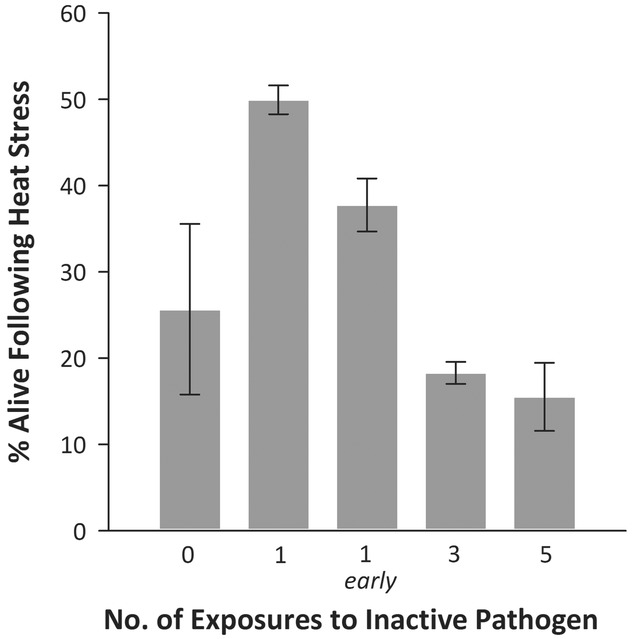
Percentage of flies surviving 20 h post heat stress following topical exposure to heat-killed fungal pathogens at different dose regimes (±SE).

We found that the influence of pathogen challenge on resistance to heat stress follows the inverted “U” dose–response pattern that is characteristic of hormesis. The number of doses of topical exposure to heat-killed spores influenced variation in resistance to heat stress (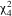 = 57.4, *P* < 0.001; Fig.1). More specifically, flies that received one dose of pathogen challenge two days before heat stress had increased resistance to heat stress compared with those that received zero (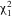 = 7.2, *P* = 0.007), three (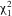 = 24.6, *P* < 0.001), or five (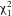 = 29.0, *P* < 0.001) doses. We also found evidence that the effect of a single dose on resistance to heat stress is temporary, as animals that received one dose 10 days prior to heat stress (1 *early*) had a nonsignificant difference in their heat stress resistance than that of untreated animals (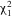 = 0.3, *P* = 0.576).

### GENOTYPE × PATHOGEN TREATMENT EFFECTS ON MORTALITY

**Figure 2:**
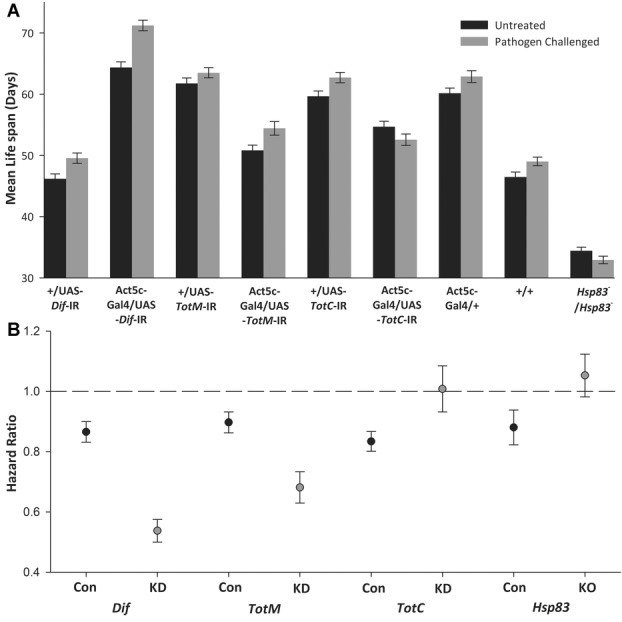
(A) Mean longevity of each genotype. Black indicates flies treated with a sham treatment (control) and gray indicates pathogen-challenged flies (±SE). Average life spans were not used in the original analysis as mortality patterns did not meet the assumptions of ANOVA investigation. (B) The influence of the topical exposure to a heat-killed fungal pathogen on the relative hazard ratio parameters to untreated flies (dashed line). Cox hazard proportions are shown for knockdown (KD) and knockout (KO) mutants in gray, and their associated pooled control genotypes (Con) in black (±SE). Values under the dashed line indicate that pathogen-challenged animals have increased survival; values above indicate that pathogen-challenged animals experience reduced survival.

We found that animals topically challenged with a single dose of heat-killed fungus generally lived longer. The pathogen challenge reduced the relative risk of death by 14% across all lines (Figs.2A, S1). In the overall model, there was evidence that longevity was influenced by pathogen treatment, genotype, and the genotype × pathogen treatment interaction (T: 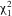 = 77.6, *P* < 0.0001; G: 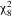 = 3099, *P* < 0.0001; G × T: 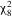 = 64.4, *P* < 0.0001; Fig.2B). It was evident that the changes in longevity in response to pathogen challenge were different in the knockdown and knockout genotypes, as removing these genotypes led to nonsignificant G × T interactions in the full model (T: 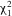 = 38.1, *P* < 0.0001; G: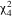 = 846, *P* < 0.0001; G × T: 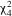 = 6.3, *P* = 0.178; Fig. S2).

The longevity benefits of pathogen challenge in isogenic (+/+) *w1118* animals depend on the expression of immunity and stress genes. While, *Dif* and *TotM* knockdown animals exhibited greater improvements in longevity in response to pathogen challenge than their associated control genotypes (G × T: 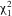 = 9.1, *P* = 0.009; 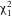 = 30.0, *P* = 0.0004, respectively; Fig.2B), *TotC* knockdown flies showed no variation in survival in response to pathogen challenge whereas their control counterparts benefited (G x T: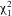 = 6.4, *P* = 0.024). There was no evidence of variation in the response to pathogen challenge on longevity in *Hsp83* knockout animals and their control genotype (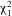 = 2.9, *P* = 0.089).

### GENOTYPE × PATHOGEN TREATMENT EFFECTS ON FECUNDITY

Across all of the lines, we found no evidence that enhanced longevity in response to pathogen challenge came with reductions in fecundity (*t*_7_ = 2.0, *P* = 0.092; Fig. S3). There was also little evidence that changes in fecundity in response to pathogen challenges were greater in the knockdowns and knockout than their associated control genotypes (G × T: *Dif*, *F*_1,323_ = 4.3, *P* = 0.156; *TotM*, *F*_1,333_ = 0.2, *P* = 0.701; *Hsp83*, *F*_1,173_ = 0.1, *P* = 0.759; *TotC*, *F*_1,334_ = 2.4, *P* = 0.126).

### TRADE-OFFS BETWEEN HORMESIS AND IMMUNITY IN OUTBRED LINES

**Figure 3:**
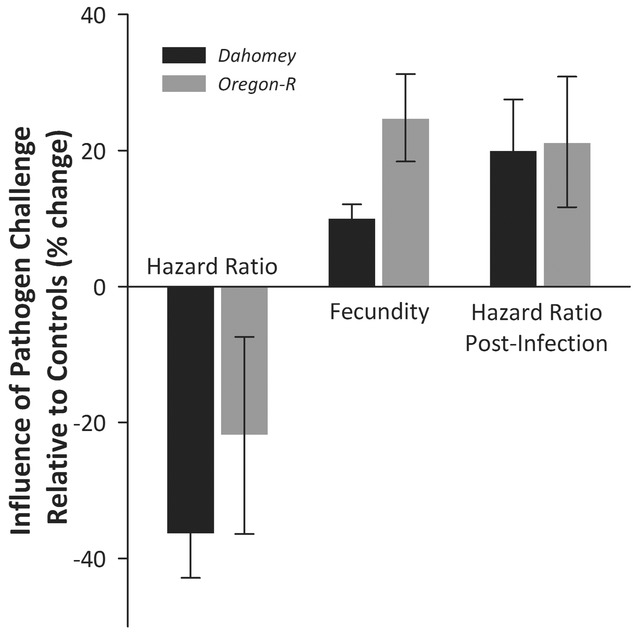
Evidence that pathogen-challenged flies experience increased survival, increased fecundity but increased susceptibility to infection in outbred lines. Estimated %change in trait values are reported ±SE. *Dahomey* line is indicated in black; *Oregon-R* line is indicated in gray.

Pathogen challenge can generate trade-offs between survival, reproduction, and immunity in outbred lines of flies. In the *Dahomey* line, we find that, in comparison to untreated animals, pathogen-challenged animals had higher survival, higher reproductive output, but also higher susceptibility to live infections (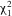 = 12.4, *P* < 0.001, *F*_1,134_ = 12.7, *P* < 0.001, 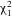 = 9.0, *P* = 0.003, respectively; Fig.3). Note that the 10% increase in fecundity resulting from pathogen challenge in the *Dahomey* line was confirmed in an independent study (*F*_1,161_ = 8.4, *P* = 0.005). The responses in the *Oregon-R* line to a pathogen challenge were similar for survival, fecundity, and susceptibility to live infection, although the survival was not significantly different (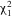 = 1.9, *P* = 0.171; *F*_1,427_ = 5.1, *P* = 0.024; 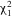 = 6.3, *P* = 0.012, respectively; see Table S1 for mean values).

## Discussion

Based on substantial documentation of hormesis (Calabrese 2005), many contemporary scientists have argued that we may be able to employ treatments that incur low-level stress as therapies for extending longevity and enhancing health (Gems and Partridge 2008; Rattan 2008; Kahn and Olsen 2009; Rattan and Demirovic 2009; Vaiserman 2010; Calabrese et al. 2012). This is an important issue to address not only for its implications for public health, but also for our understanding of life-history evolution (Forbes 2000; Costantini et al. 2010). Our study shows that there can be immunological costs for treatments that extend life.

Although the induction of hormesis by topical challenge with a dead pathogen may seem unusual, our findings are similar to other described cases of pathogen-induced improvements in physiology (Polak and Starmer 1998; Chadwick and Little 2005; Leroy et al. 2012; Papp et al. 2012; Ermolaeva et al. 2013). The 18% average decrease in the hazard ratio of pathogen-challenged animals observed across all wild-type and control genotype lines is also comparable to the beneficial influences of other stress treatments on longevity in fruit flies, including 5% and 10% from heat stress (Khazaeli et al. 1997; Hercus et al. 2003), 15% from hypergravity (Le Bourg and Minois 1997; Le Bourg et al. 2000); 30% from spermidine (Eisenberg et al. 2009), and 13% and 9% from cold stress (Le Bourg 2007; Le Bourg 2012).

Most of the previous work on the life-extending properties of hormesis has focused on phenomenology, that is, how, but not why, organisms benefit from stress (Forbes 2000; Gems and Partridge 2008; Rattan 2008; Calabrese et al. 2012, although see Costantini et al. 2010). Our results are consistent with the idea that animals shift their life histories in response to environmental stress (Tatar et al. 2003). We found that topical exposure of dead fungal spores changes a number of key life-history traits and that immune and stress gene expression in the host alters the longevity benefits of the pathogen challenge. We also documented hormetic responses in both genetically mutant isogenic lines and outbred laboratory lines, which indicates that hormesis is not simply an artifact of inbreeding (Liao et al. 2010; Nakagawa et al. 2012). Our findings provide clear evidence that stress genes facilitate and immune genes suppress hormesis, which is in line with previous studies of the genetic basis of hormesis (Kristensen et al. 2003; Mattson 2010; Calabrese et al. 2012; Le Bourg et al. 2012; Gartner and Akay 2013).

It is not clear whether mild exposure to stressors enhances Darwinian fitness or alters one aspect of fitness at a cost to another (Forbes 2000; Costantini et al. 2010). There are a handful of studies that indicate that hormetic benefits on longevity are temporary (Wu et al. 2008) and come with trade-offs in fecundity (Maynard Smith 1958; Krebs and Loeschcke 1994; Lane et al. 1996; Markowska 1999; Le Bourg et al. 2000; Sørensen et al. 2007). But, many studies show that mild exposure to stressors can simultaneously improve survival and fecundity (see Costantini et al. 2010) and it is often presumed that hormetic benefits are cost free (Rattan and Demirovic 2009; Calabrese et al. 2012). Our findings are consistent with the trade-off explanation for hormesis. We found that hormetic responses to stress were greater in animals lacking expression of immune genes *Dif* and *TotM*, which we had previously established provide protection against direct or sexually transmitted fungal infections, respectively (Zhong et al. 2013). Additionally, we found that although pathogen challenge simultaneously increases survival and fecundity, it leads to trade-offs with immunity. These results mirror the finding that the activation of transcription factor SKN-1 in nematodes enhances resistance to oxidative stress and longevity, but increases susceptibility to infection (Papp et al. 2012).

Studies of immune priming have reported that both dead and live pathogen challenges increase, not decrease, susceptibility to subsequent infections (Lawniczak et al. 2007; Pham et al. 2007; Roth et al. 2009). Although our findings seem to be at odds with this result, it might be plausible that immune priming and hormesis might represent divergent strategies for fighting off infections. When they are infected with a lethal pathogen, it is known that animals adopt a myriad of life-history strategies, including fecundity reduction, a long-term strategy that reduces reproductive output and increases resistance (Hurd 2001), and fecundity compensation, a terminal strategy that temporarily increases reproductive output and decreases immune function (Velando et al. 2006; Weil et al. 2006). It is interesting to note that the dietary and temperature conditions in which we carried the current study are identical to those that lead to a terminal, fecundity compensation strategy in flies infected with a live fungus (V. L. Hunt et al., unpubl. ms.). Thus, it seems plausible that pathogen-induced hormesis might have occurred in our study because flies are mounting a terminal strategy to an infection that never comes.

An alternative explanation for our findings is that hormesis is an artifact of domestication and/or laboratory adaptation (Nakagawa et al. 2012). As we only employed laboratory-selected lines and strains based on isogenic laboratory stocks, our study was not designed to test this question; however, whether hormesis only occurs in domesticated animals does not detract from our findings. The key point is that, when it occurs, hormesis leads to trade-offs with other fitness traits.

Our findings do not necessarily imply that we should ban low-level stress treatments as therapies for human health. It seems quite plausible that in healthy patients, we could employ our natural life-history responses to environmental cues to further improve their health. However, the consequences of hormetic treatments for infected patients could be dire. It is clear that the immunological trade-offs of hormesis need to be identified, acknowledged, and explicitly tested, as others have stated (Gems and Partridge 2008; Rattan and Demirovic 2009; Vaiserman 2010; Calabrese et al. 2012). Further studies of hormesis in humans and model systems could eventually help us identify the selective forces and molecular mechanisms that underlie life-history constraints.

## DATA ARCHIVING

The doi for our data is 10.5061/dryad.85nn2.

## Supporting Information

Figure S1 Cox hazard proportions of pathogen-challenged flies in relation to their untreated counterparts (dashed line) for each genotype (±SE).

Figure S2 Survival curves for each of the nine genotypes.

Figure S3 Mean fecundity of each genotype.

Table S1 Response of trait expression to exposure to heat-killed fungal spores (pathogen challenged) and untreated (control) conditions from the wild-type outbred strains *Dahomey* and *Oregon-R*.
