# Supplementary material for: HORMESIS RESULTS IN TRADE-OFFS WITH IMMUNITY
Source: Evolution. 2014 Jun 20;68(8):2225–33. doi: 10.1111/evo.12453 (PMC4282086; doi:10.1111/evo.12453)

Supplementary Figure 1: Cox hazard proportions of pathogen challenged flies in relation to their untreated counterparts (dashed line) for each genotype (± SE). Black circles indicate background genotypes and grey bars represent knockdown and knockout lines. ** - *P* < 0.01, *** - *P* < 0.001

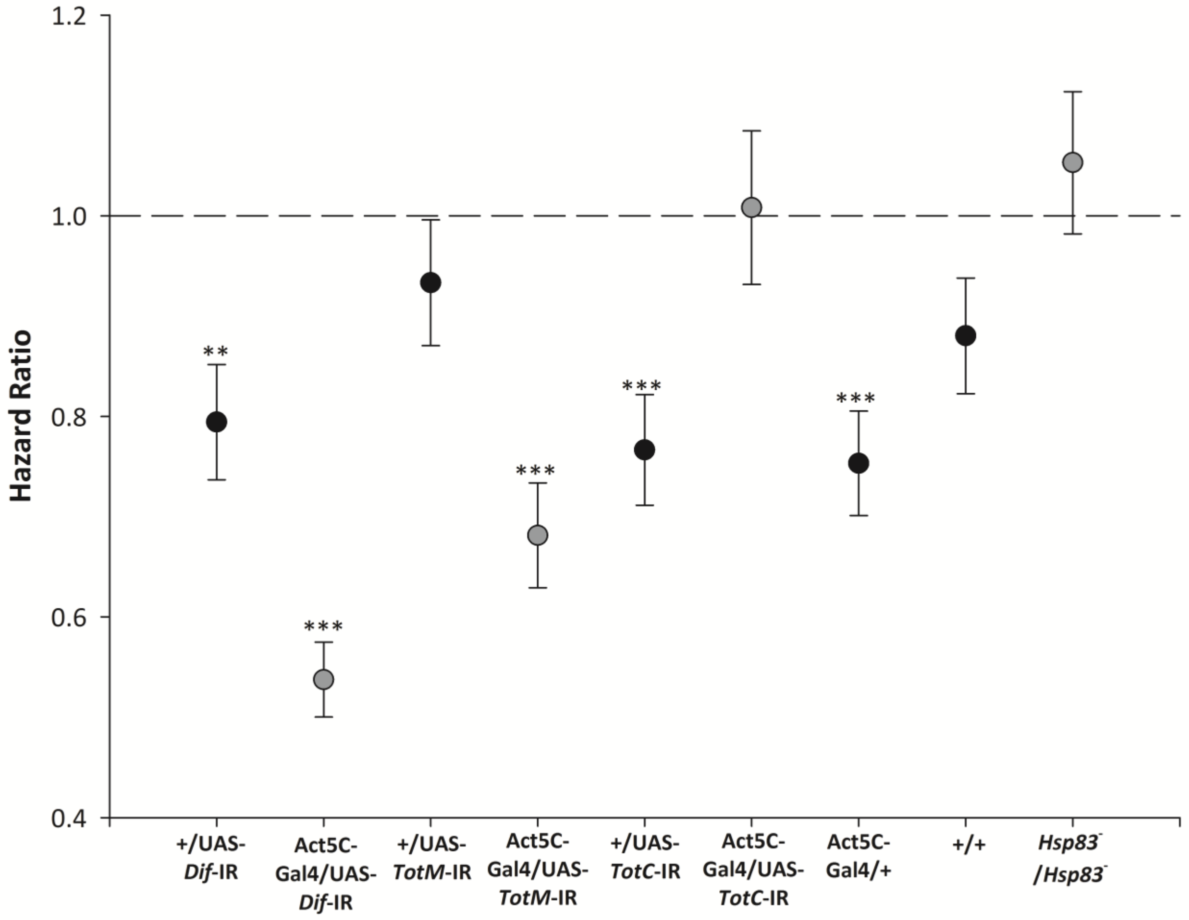

Supplement: Figure S1 — Cox hazard proportions of pathogen-challenged flies in relation to their untreated counterparts (dashed line) for each genotype (±SE). [file evo0068-2225-SD1.pdf]
