# Supplementary material for: HORMESIS RESULTS IN TRADE-OFFS WITH IMMUNITY
Source: Evolution. 2014 Jun 20;68(8):2225–33. doi: 10.1111/evo.12453 (PMC4282086; doi:10.1111/evo.12453)

Supplementary Figure 2: Survival curves for each of the 9 genotypes. Black lines are the survival curves for untreated cohorts while grey lines represent survival curves for pathogen challenged cohorts.

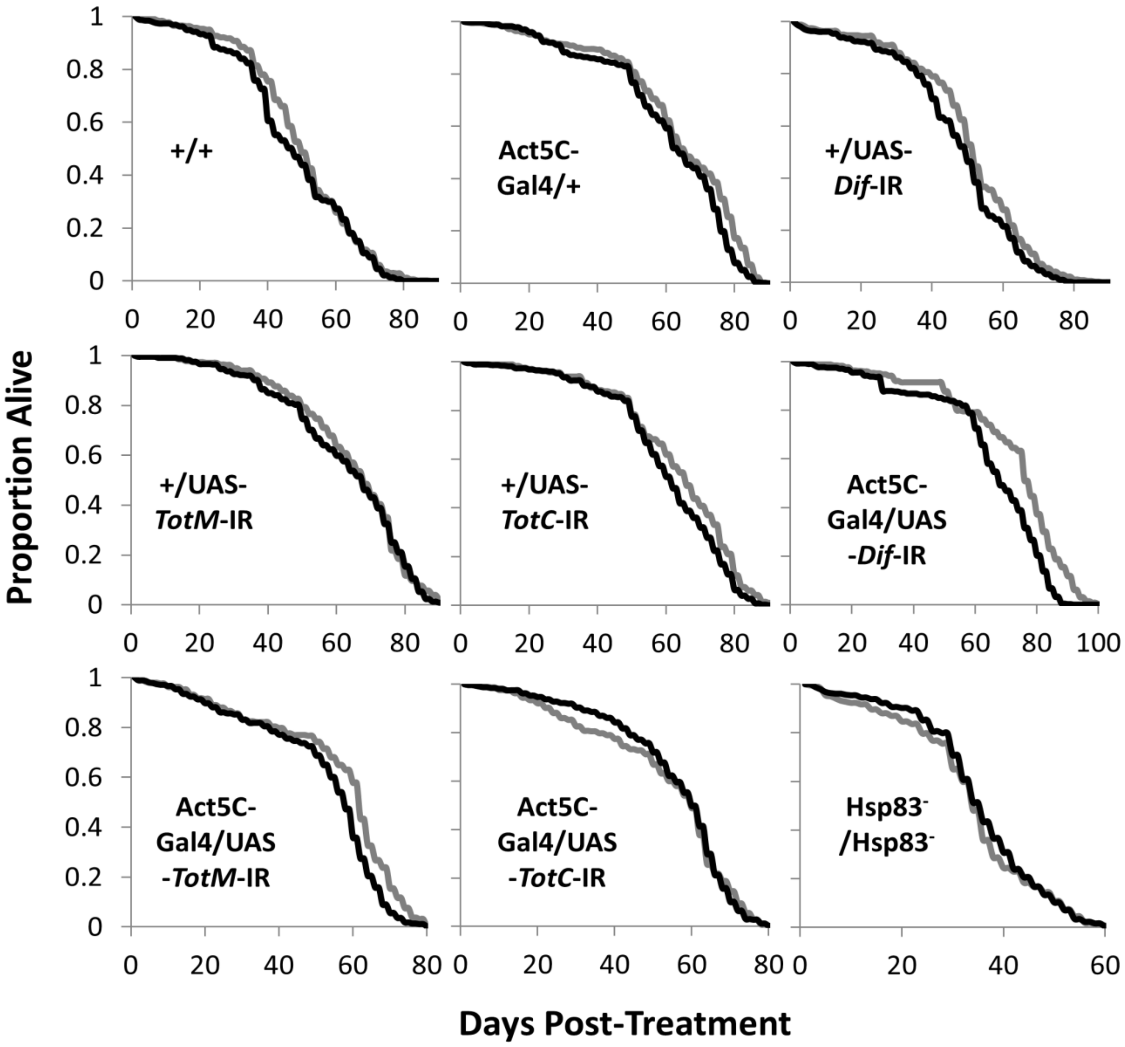

Supplement: Figure S2 — Survival curves for each of the nine genotypes. [file evo0068-2225-SD2.pdf]
