# Supplementary material for: HORMESIS RESULTS IN TRADE-OFFS WITH IMMUNITY
Source: Evolution. 2014 Jun 20;68(8):2225–33. doi: 10.1111/evo.12453 (PMC4282086; doi:10.1111/evo.12453)

Supplementary Figure 3: Mean fecundity of each genotype. Black indicates flies treated with a sham treatment (control) and grey indicates flies treated with heat-killed fungal spores (± SE). Average fecundity was taken from pupae counts over 10 day samples. * - $P < 0.05$, ** - $P < 0.01$

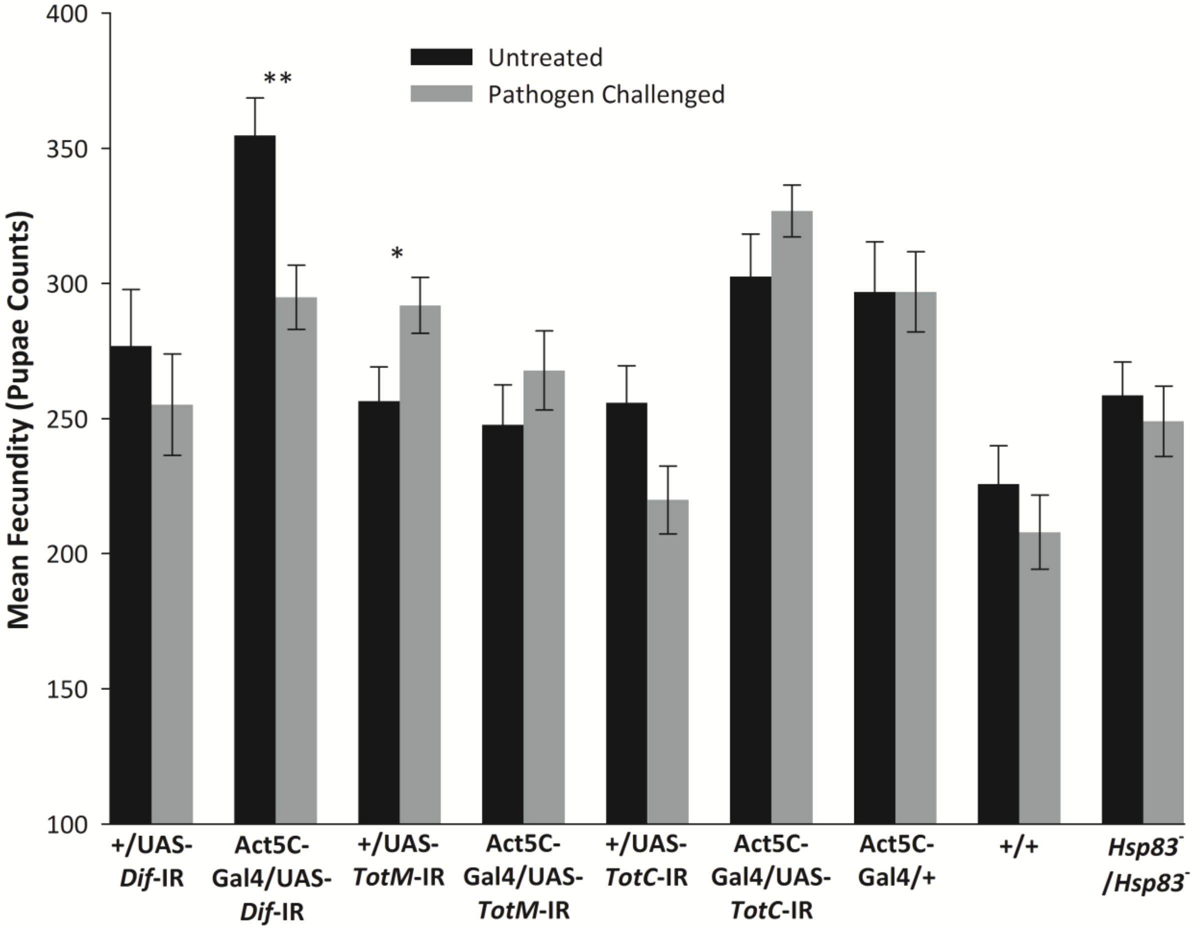

Supplement: Figure S3 — Mean fecundity of each genotype. [file evo0068-2225-SD3.pdf]
