# Supplementary material for: HORMESIS RESULTS IN TRADE-OFFS WITH IMMUNITY
Source: Evolution. 2014 Jun 20;68(8):2225–33. doi: 10.1111/evo.12453 (PMC4282086; doi:10.1111/evo.12453)
Supplement: Table S1 — Response of trait expression to exposure to heat-killed fungal spores (pathogen challenged) and untreated (control) conditions from the wild-type outbred strains Dahomey and Oregon-R. [file evo0068-2225-SD4.docx]

**Supplementary Table 1:** Response of trait expression to exposure to heat-killed fungal spores (pathogen challenged) and untreated (control) conditions from the wild-type outbred strains *Dahomey* and *Oregon-R*. Mean lifespans are given for both uninfected and infected conditions. Mean fecundity under uninfected conditions are shown (± SE).

***Dahomey Oregon-R***

**Trait Control Pathogen Challenged Control Pathogen Challenged**

Lifespan 36.3 (± 0.9) 39.7 (± 0.9) 38.0 (± 2.0) 41.3 (± 2.0)

(Post-treatment)

Fecundity 217.6 (± 3.9) 239.2 (± 4.6) 31.0 (± 2.3) 38.8 (± 2.5)

Lifespan 6.9 (± 0.3) 6.3 (± 0.2) 7.4 (± 0.5) 6.5 (± 0.4)

(Post-infection)
